# Association of Ticagrelor Metabolic SNPs With Adverse Drug Reactions in Patients With Acute Coronary Syndrome

**DOI:** 10.1002/clc.70232

**Published:** 2025-12-11

**Authors:** Yanming Zhang, Gaoxiu Yu, Cong Wang, Jingwen Song, Junmei Liu, Feng Chen

**Affiliations:** ^1^ Department of Cardiology, Changhai Hospital Naval Military Medical University Shanghai Yangpu People's Republic of China

## Abstract

**Background:**

Dual antiplatelet therapy with aspirin and a P2Y₁₂ inhibitor is standard for patients with acute coronary syndrome (ACS) undergoing percutaneous coronary intervention. While ticagrelor offers superior efficacy in reducing ischemic events compared to clopidogrel, its use is limited by a higher incidence of dyspnea, an adverse reaction whose underlying predictors remain incompletely understood. Genetic variations in CYP3A4/5, the principal enzymes responsible for ticagrelor metabolism, may influence interindividual susceptibility to this side effect.

**Methods:**

In a prospective cohort of 385 ACS patients on ticagrelor, we genotyped CYP3A4 rs2242480 and CYP3A5 rs776746. Outcomes (dyspnea per CTCAE v5.0, bleeding per BARC criteria) were assessed over 1 year. Associations were analyzed using logistic regression and GMDR modeling.

**Results:**

The CYP3A5 rs776746 genotype strongly predicted dyspnea risk. Compared to the CC genotype, CT and TT genotypes were associated with a 55% and 91% reduced risk, respectively. Carriers of the combined CT/TT genotypes had a 63% lower risk. CC genotype carriers (poor metabolizers) exhibited a 2.3‐fold higher dyspnea incidence. No significant associations were found for CYP3A4 rs2242480 or for bleeding outcomes.

**Conclusion:**

The CYP3A5 rs776746 CC genotype is a significant genetic biomarker for ticagrelor‐induced dyspnea. Pre‐emptive genotyping could enable personalized antiplatelet therapy, such as alternative P2Y₁₂ inhibitors for high‐risk CC carriers, to improve patient safety.

## Graphical abstract

The CYP3A5 rs776746 CC genotype is a strong biomarker for ticagrelor‐induced dyspnea, conferring a 2.3‐fold higher risk. Genotype‐guided antiplatelet therapy may mitigate this adverse reaction, enabling personalized treatment for ACS patients.

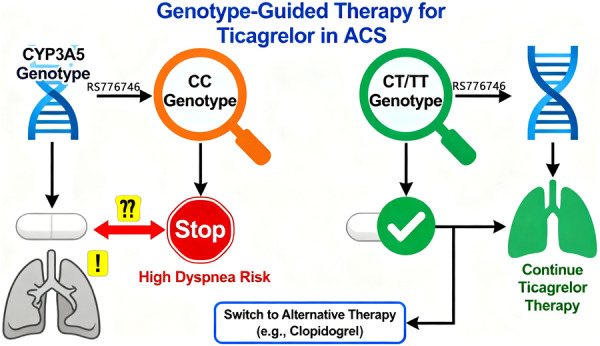

## 1 Introduction

The 2025 American College of Cardiology (ACC) updated Clinical Practice Guideline for the Management of Acute Coronary Syndromes provides evidence‐based recommendations on antithrombotic regimens following revascularization, grounded in data from multicenter randomized controlled trials. The guideline specifically emphasizes that for acute coronary syndrome (ACS) patients undergoing percutaneous coronary intervention (PCI), a dual antiplatelet therapy (DAPT) strategy comprising aspirin and ticagrelor is associated with a significantly lower risk of all‐cause mortality compared to a clopidogrel‐based regimen [1, 2]. Furthermore, this strategy reduces the incidence of major adverse cardiovascular events (MACE) by 16% and lowers the risk of stent thrombosis recurrence. Consequently, this recommendation has been elevated to a Class I recommendation (Level of Evidence: A), underscoring the superior long‐term prognostic benefit of this treatment approach. The ticagrelor group exhibited a lower combined incidence of cardiovascular death, myocardial infarction, and stroke compared to the clopidogrel group, however, it had a higher incidence of adverse events such as bleeding, dyspnea, and increased uric acid levels. Within the metabolic pathway of ticagrelor, the cytochrome P450 isoenzymes CYP3A4/5 serve as the key catalytic enzymes responsible for mediating its conversion to AR‐C124910XX, the pharmacologically active intermediate metabolite. Pharmacodynamic (PD) studies demonstrate that this active metabolite exhibits a high degree of similarity to the parent drug, ticagrelor, in terms of P2Y12 receptor inhibitory potency. Furthermore, clinical observations confirm that both entities demonstrate equivalence in the intensity and duration of platelet aggregation inhibition (with maximum platelet aggregation inhibition rates > 90% for both). This bioactivation pathway underpins the central role of AR‐C124910XX in sustaining the overall antithrombotic efficacy of ticagrelor [3]. The plasma concentrations of ticagrelor and its active metabolite, AR‐C124910XX, are significantly dependent on the efficiency of CYP3A4/5‐mediated biotransformation. Consequently, their pharmacokinetic profile exhibits genotype‐dependent interindividual variability. Specifically, genetic polymorphisms (single nucleotide polymorphisms, SNPs) in the genes encoding CYP3A4/5 may lead to variations in enzymatic activity expression levels [4]. Pharmacodynamic studies have demonstrated a significant positive correlation between the systemic exposure of ticagrelor's active metabolite and the incidence of ticagrelor‐associated dyspnea. Furthermore, the intrinsic metabolic processing of ticagrelor within the body may be a key determinant influencing the occurrence of ticagrelor‐related adverse reactions [5]. Accurate identification of CYP3A4/5 genetic polymorphisms holds considerable practical significance for implementing clinical precision medicine, particularly regarding medication safety and adverse drug reactions (ADRs) in ACS patients. Currently, research on ticagrelor dose individualization in ACS patients reveals a notable gap. This study delves into the relationship between CYP3A4/5 genetic polymorphisms and ticagrelor‐associated ADRs in ACS patients at the level of genetic polymorphism research.

## 2 Materials and Methods

### 2.1 General Information

This study enrolled patients with Acute Coronary Syndrome (ACS) (diagnosed according to the 2019 Chinese Guidelines for the Emergency Diagnosis and Treatment of ACS) who underwent Percutaneous Coronary Intervention (PCI) at Chang Hai Hospital, Naval Medical University between January and December 2022. All participants were informed of the details of each test indicator prior to the study and provided written informed consent. This study was approved by the Hospital Ethics Committee (Ethics Approval No.: SK2020‐044).

Inclusion Criteria: ① PCI indication met the 2022 Chinese PCI Guideline criteria, with complete surgical records. ②Post‐procedural standard dual antiplatelet therapy (DAPT) regimen: Ticagrelor (180 mg loading dose followed by 90 mg twice daily maintenance) combined with Aspirin (100 mg once daily). ③Assessable medication adherence (categorized as fully adherent, partially adherent, or non‐adherent).

Exclusion Criteria: ① Use of other antiplatelet or anticoagulant medications.② Severe underlying diseases, including:Heart failure (New York Heart Association [NYHA] functional class III or IV). Left ventricular ejection fraction (LVEF) < 30%. Chronic obstructive pulmonary disease (COPD) (Global Initiative for Chronic Obstructive Lung Disease [GOLD] stage ≥ 3). Sleep apnea (Apnea‐Hypopnea Index [AHI] ≥ 30 events per hour). Requirement for supplemental oxygen therapy. ③ Comorbidities including: Atrial fibrillation (with CHA₂DS₂‐VASc score ≥ 2). Active malignancy. Life expectancy < 1 year. ④ Concurrent use of medications known to cause dyspnea (Angiotensin‐Converting Enzyme Inhibitors [ACEIs], Angiotensin II Receptor Blockers [ARBs], opioids, theophylline derivatives, strong CYP3A4/5 inhibitors) or long‐term use of Non‐Steroidal Anti‐Inflammatory Drugs (NSAIDs) or corticosteroids. ⑤High bleeding risk, defined by: Coagulopathy. Active bleeding. Thrombocytopenia. ⑥ Inability to complete the follow‐up period.

### 2.2 Reagents

Whole Blood Genomic DNA Extraction Kit: Tiangen Biotech (Beijing) Co. Ltd., China. BigDye™ Terminator v3.1 Cycle Sequencing Kit: Thermo Fisher Scientific, USA. DNA Gel Extraction Kit: Axygen Biosciences (Corning Incorporated), USA. DNase/RNase‐Free Microcentrifuge Tubes: Corning Incorporated, USA. Absolute Ethanol: Sinopharm Chemical Reagent Co. Ltd., China. SYBR Green Quantitative PCR Master Mix: Accurate Biology (Hunan) Co. Ltd., China. 2× Taq Master Mix (Dye Plus): Novoprotein Scientific & Technology Co. Ltd., China.

### 2.3 Major Equipment

DNA Sequencer: ABI 3730xl Genetic Analyzer, Applied Biosystems (Thermo Fisher Scientific), USA. Real‐Time PCR System: LightCycler® 480 Instrument II, Roche Diagnostics, Switzerland. Laminar Flow Cabinet: SW‐CJ Series Clean Bench, Shanghai Zupei Biotechnology Co. Ltd., China. Microvolume Spectrophotometer: NanoDrop™ One/OneC, Thermo Fisher Scientific, USA. Thermal Cycler: TP600 PCR System, Takara Bio Inc., Japan. Refrigerated Microcentrifuge: Sigma 3‐18KS or similar model, Sigma‐Aldrich (Merck KGaA), USA/Germany. Ultra‐Low Temperature Freezer: SANYO (Panasonic Healthcare) MDF‐U73V or similar ‐86°C Upright Freezer, SANYO Electric Co. Ltd. (now part of Panasonic), Japan.

### 2.4 Gene Locus Selection

Functional SNPs were selected using a multi‐stage strategy. Initially, all SNPs within the CYP3A4 and CYP3A5 gene regions were retrieved from the Ensembl database. Subsequent stepwise filtering was applied based on the following criteria:

①Priority inclusion of SNPs exhibiting high frequency within East Asian populations (specifically Han Chinese subgroups CHB/CHS) in the 1000 Genomes Project and the China Metabolic Analytics Project (ChinaMAP) database.

② Restriction to SNPs located within the coding regions of CYP3A4 and CYP3A5 genes, or within their putative regulatory regions (±10 kb).

③ Minimum Allele Frequency (MAF) requirement > 25%.

④ Selection of tagSNPs that have been previously reported to show significant associations with drug metabolism, efficacy, or adverse drug reactions.

Ultimately, two single nucleotide polymorphisms (SNPs) were selected for study: CYP3A4 rs2242480 and CYP3A5 rs776746. Functional validation studies demonstrated that proteins binding to these loci influence gene expression.

### 2.5 Distribution Characteristics of CYP3A4/5 Genetic Polymorphisms

This study retrieved genetic polymorphism data for European, East Asian, African, and American populations from Phase 3 of the 1000 Genomes Project database through the National Center for Biotechnology Information (NCBI). Comparative analysis revealed that the allele frequencies at rs776746 and rs2242480 in our study cohort closely resembled those of the East Asian population (Table 1).

**TABLE 1:** Comparison of CYP3A4/5 genetic polymorphism distribution across ethnic populations.

	African population	American population	East Asian population	European population	Study cohort
rs776746					
C	0.180	0.797	0.713	0.943	0.705
T	0.820	0.203	0.287	0.057	0.295
rs2242480					
C	0.150	0.607	0.732	0.918	0.750
T	0.850	0.393	0.268	0.082	0.250

### 2.6 General Clinical Data and Collected Parameters

Patients were followed for 1 year. Clinical data and parameters were systematically collected, encompassing the following categories: Baseline Characteristics: Name, Hospitalization ID, Sex, Age (years at enrollment), Height (cm), Weight (kg), Body Mass Index (BMI, kg/m²), Ethnicity, Contact information, Lifestyle habits. Disease and Treatment Information: Diagnostic details, Medication record (including study drug), Concomitant medications. Laboratory and Imaging Parameters: Coagulation profile, Complete blood count (CBC), Liver and kidney function tests, Heart failure biomarkers, Imaging studies.

### 2.7 Follow‐Up Protocol

To comprehensively evaluate safety profiles and associated influencing factors, patients with Acute Coronary Syndrome (ACS) underwent a 1‐year prospective follow‐up observation after hospital discharge. A standardized follow‐up questionnaire was uniformly administered to all participants to ensure data standardization and completeness. Key aspects included:

Follow‐up Schedule and Modality: Follow‐up Visits: Systematically conducted at 1, 3, 6, and 12 months post‐discharge. Unscheduled Visits: Immediately arranged if patients reported suspected endpoint events (e.g., new/worsening chest pain, dyspnea).

Follow‐up Procedures: Clinic Visits: Performed by cardiologists. Included: Physical examination; Laboratory testing; Repeat imaging studies; Telephone Follow‐up: Employed for patients unable to attend clinic visits. Utilized a structured questionnaire to collect information on: Symptoms; Medication adherence; Adverse events.

### 2.8 Outcome Measures

Primary Outcome: Dyspnea graded per CTCAE v5.0 (Grade ≥ 1 defined as event): Grade 1: Mild (worsened by exertion); Grade 2: Moderate (limiting ADL); Grade 3: Severe (requiring O₂); Grade 4: Life‐threatening; Grade 5: Death.

Secondary Outcomes: Bleeding Events: Classified by BARC criteria (Type ≥ 3 defined as significant): Type 3a: Hb drop ≥ 3 g/dL or transfusion; Type 3b/3c: Severe; Type 4: CABG‐related; Type 5: Fatal. Repeat Revascularization: Repeat PCI for recurrent ischemia or in‐stent restenosis. All‐Cause Mortality: Death from any cause.

### 2.9 Adverse Drug Reaction (ADR) Assessment Methodology

For causality assessment of adverse drug reactions (ADRs), this study employed the Karch‐Lasagna algorithm, an authoritative method in the field. This algorithm employs scientifically rigorous criteria to precisely categorize the causal relationship between a drug and an observed adverse event into five distinct levels: definite, probable, possible, conditional, and doubtful.

The assessment mandates the following conditions: Temporal Relationship: The onset of the ADR must follow drug administration within a plausible and reasonable timeframe. Symptom Profile: The observed ADR must be highly concordant with the known adverse reaction profile of the drug. Dechallenge: The ADR should resolve upon discontinuation of the drug. Rechallenge: Reappearance of the ADR upon re‐exposure to the drug is required for a definite causal relationship.

Within the context of this study, the attribution of specific ADRs potentially related to ticagrelor therapy—including but not limited to respiratory events (e.g., dyspnea, chest tightness) and hemorrhagic events (e.g., gastrointestinal hemorrhage, gingival bleeding)—was strictly adjudicated using the Karch‐Lasagna algorithm. An ADR was attributed to ticagrelor only if the causality assessment reached the “definite or probable” level based on a comprehensive evaluation.

### 2.10 Genetic Analysis

Genomic DNA was isolated from EDTA‐anticoagulated venous blood samples using protease K lysis and column purification (TIANamp Blood DNA Kit). DNA purity (A260/A280 ratio 1.8–2.0) and concentration (≥ 1 ng/μL) were verified by UV spectrophotometry. Target regions were amplified via quantitative real‐time PCR (qPCR) using SYBR Green Pro Taq HS Premix under the following conditions: 95°C for 5 min; 40 cycles of 95°C for 10 s, 60°C for 10 s, 72°C for 10 s. PCR products were verified by 2% agarose gel electrophoresis, gel‐purified, and re‐amplified. Final products were purified by ethanol precipitation and sequenced using capillary electrophoresis (ABI 3730xl DNA Analyzer). Genotype data were generated using Sequencing Analysis v6.0 software.

### 2.11 Statistical Methods

Statistical analyses were performed using SPSS software (version 25.0). Categorical variables are presented as frequency (percentage). Group comparisons for categorical data were conducted using chi‐square tests or Fisher's exact tests, as appropriate. Continuous variables are expressed as mean ± standard deviation for normally distributed data (analyzed using independent samples *t*‐tests or paired *t*‐tests) or as median (interquartile range) for non‐normally distributed data (analyzed using Mann‐Whitney U tests or Wilcoxon signed‐rank tests).

Multivariable logistic regression was employed to calculate odds ratios (ORs) with corresponding 95% confidence intervals (CIs). The threshold for statistical significance was set at α = 0.05 (two‐tailed). Multiple testing corrections were applied using the Bonferroni method where appropriate. Normality was verified for all analyses to ensure methodological appropriateness.

### 2.12 Basic Information Regarding the Research Process and Cohort

**FIGURE 1:**
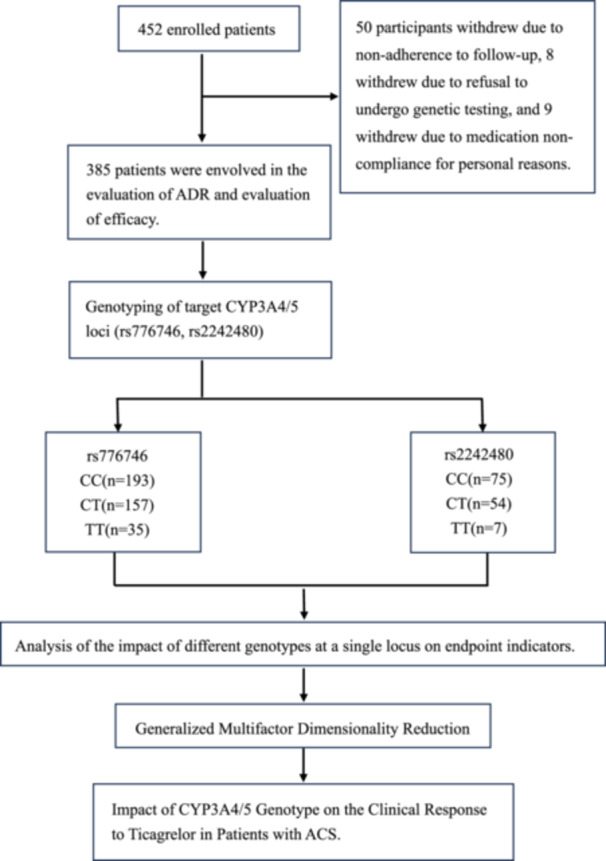
Research flow diagram.

The initial screening cohort of this study comprised 452 patients who fulfilled the inclusion criteria. During the study, 50 patients withdrew due to non‐compliance with follow‐up, eight withdrew due to refusal of genetic testing, and nine withdrew due to irregular medication administration for personal reasons. Ultimately, 385 patients successfully completed the entire process of case and indicator evaluation for standardized dual antiplatelet therapy plus genotyping over a 12‐month period. For detailed information, please refer to the enrollment process diagram (Figure 1).

## 3 Result

### 3.1 Comparison of Clinical Characteristics Between Genotype Groups During Follow‐Up

Results (Table 2) comparing the baseline characteristics and clinical outcomes between carriers of the rs2242480 (*n* = 136) and rs776746 (*n* = 385) loci demonstrated homogeneity in core demographic parameters between the two groups. No statistically significant differences (*p* > 0.05) were observed in the proportion of males (78.68% vs. 81.82%), age (64.5 ± 11.4 years vs. 63.6 ± 12.6 years), body mass index (24.97 ± 2.80 vs. 25.49 ± 7.38 kg/m²), or smoking rate (53.7% vs. 44.9%).

**TABLE 2:** Clinical characteristics of follow‐up in study population.

Variable	rs2242480 (*n* = 136)	rs774746 (*n* = 385)	χ^2^, Z/t	*P*
Demographics				
Male, %	107 (78.68)	315 (81.82)	0.644	0.422
Age, y	64.53 ± 11.44	63.60 ± 12.55	0.759	0.448
BMI, kg/m^2^	24.97 ± 2.80	25.49 ± 7.38	−0.81	0.418
Clinical parameters				
Smoking, %	73 (53.7)	173 (44.9)	3.081	0.079
Hypertension, %	93 (68.4)	252 (65.5)	0.388	0.535
Diabetes, %	36 (26.5)	99 (25.7)	0.030	0.863
BNP, pg/mL	156.17 (45.9, 350.3)	133.05 (43.9, 332.8)	−0.634	0.526
PT, s	12.57 ± 1.53	12.54 ± 1.52	−0.261	0.794
TT, s	16.05 ± 1.87	16.03 ± 1.90	−0.634	0.527
APTT, s	30.02 ± 4.97	29.79 ± 4.65	−0.481	0.631
FIB, g/L	3.43 ± 0.78	3.50 ± 0.79	0.813	0.417
D‐Dimer, mg/L	0.11 (0.05, 0.26)	0.11 (0.06, 0.25)	−1.41	0.159
Cr, μmol/L	83.00 (70.30, 98.00)	78.00 (66.00, 92.00)	−2.508	0.012
Hb, g/L	137.43 ± 14.77	138.26 ± 15.90	0.530	0.596
PLT,/L	211.31 ± 49.36	215.15 ± 49.69	0.775	0.438
LDL‐C, mmol/L	1.58 ± 0.79	1.60 ± 0.80	0.335	0.737
ALT, U/L	31.09 ± 8.02	32.02 ± 7.98	1.167	0.224
AST, U/L	29.95 ± 8.71	29.35 ± 8.07	−0.734	0.764
Genotype distribution			2.459	0.292
CC, %	75 (55.15)	193 (50.13)	——	——
CT, %	54 (39.71)	157 (40.78)	——	——
TT, %	7 (5.15)	35 (9.09)	——	——
CT + TT, %	61 (44.85)	192 (49.87)	1.013	0.314
Follow‐up events^a^				
Bleeding events, %	6 (4.41)	13 (3.38)	0.306	0.580
Revascularization, %	10 (7.35)	26 (6.75)	0.056	0.813
Dyspnea, %	25 (18.38)	71 (18.44)	0.00	0.988
All‐cause mortality, %	8 (5.88)	15 (3.90)	0.940	0.332

a Follow‐up events include bleeding events, revascularization, dyspnea, and all‐cause mortality.

Among the biochemical markers analyzed, only serum creatinine concentration showed a significant between‐group difference (median value 83.00 μmol/L in the rs2242480 group vs. 78.00μmol/L in the rs776746 group, *p* = 0.012). No significant differences (*p* > 0.05) were found in B‐type natriuretic peptide (BNP) levels, coagulation parameters, D‐dimer levels, or low‐density lipoprotein cholesterol (LDL‐C) levels.

Analysis of genotype distributions revealed no significant differences (*p* = 0.292–0.314) in the frequencies of the CC, CT, and TT genotypes, or in the combined CT/TT proportion between the two groups.

The incidence rates of clinical endpoint events during follow‐up were consistent between groups and did not reach statistical significance (*p* > 0.05) for any event: bleeding events (4.41% vs. 3.38%), coronary revascularization (7.35% vs. 6.75%), dyspnea symptoms (18.38% vs. 18.44%), or all‐cause mortality (5.88% vs. 3.90%).

To ensure sufficient statistical power for the primary hypothesis, full sample analysis for this locus (*n* = 385) was prioritized. Given that early data for CYP3A4 (rs2242480) showed no independent association (*p* > 0.05), it was categorized as exploratory. Consequently, in‐depth statistical analysis for this locus was performed on a subset of samples (*n* = 136) to conserve sequencing resources and time.

### 3.2 Analysis of Dyspnea Outcomes by Genetic Locus

rs776746 Locus Analysis: Univariate and multivariable logistic regression analyses for the rs776746 locus are presented in Table 3. Univariate analysis revealed that both the CT genotype (OR = 0.45, 95% CI = 0.26–0.80, *p* = 0.006) and the TT genotype (OR = 0.09, 95% CI = 0.01–0.65, *p* = 0.017) were significantly associated with a reduced risk of dyspnea. In the multivariable analysis, the CT genotype (OR = 0.44, *p* = 0.005) and TT genotype (OR = 0.08, *p* = 0.014) remained significantly protective, indicating that the genotype effect is independent of other covariates. Hypertension was associated with an increased risk of dyspnea in the univariate analysis (OR = 1.86, *p* = 0.040) but was not included in the final multivariable model, suggesting its association may not persist after adjustment or could be confounded by genotype effects.

**TABLE 3:** Analysis of univariate and multivariable logistic regression for dyspnea at the rs776746 locus.

Variable	Univariate analysis OR (95% CI)	*P*	Multivariate analysis OR (95% CI)	*P*
Age, y	1.02 (0.99–1.03)	0.258	1.02 (0.99–1.04)	0.133
Female, %	1.44 (0.70–3.00)	0.199	——	——
BMI, kg/m2	0.97 (0.89–1.05)	0.430	——	——
Smoking, %	1.08 (0.64–1.81)	0.772	——	——
Hypertension, %	1.86 (1.00–3.36)	0.040	——	——
Diabetes, %	0.73 (0.39–1.37)	0.329	——	——
Cr, μmol/L	1.00 (0.99–1.01)	0.820	——	——
Genotype distribution				
CT	0.45 (0.26 – 0.80)	0.006	0.44 (0.25–0.78)	0.005
TT	0.09 (0.01–0.65)	0.017	0.08 (0.01–0.59)	0.014
CT + TT	0.37 (0.20–0.68)	0.001	——	——
BNP, pg/mL	1.00 (1.00–1.00)	0.355	——	——
Hb, g/L	0.99 (0.98–1.01)	0.501	——	——
PLT,/L	1.00 (0.99–1.00)	0.435	——	——
LDL‐C, mmol/L	0.95 (0.69–1.31)	0.755	——	——
ALT, U/L	0.98 (0.95–1.01)	0.241	——	——
AST, U/L	1.00 (0.97–1.04)	0.877	——	——

rs2242480 Locus Analysis: Results of univariate and multivariable logistic regression analyses for the rs2242480 locus are shown in Table 4. Neither the univariate nor the multivariable analysis demonstrated a significant association between genotype and dyspnea risk (*p* > 0.05 for all comparisons).

**TABLE 4:** Analysis of univariate and multivariable logistic regression for dyspnea at the rs2242480 locus.

Variable	Univariate analysis OR (95% CI)	*P*	Multivariate analysis OR (95% CI)	*P*
Age, y	1.01 (0.97–1.05)	0.513	——	——
Female, %	0.45 (0.12–1.61)	0.217	——	——
BMI, kg/m^2^	1.00 (0.86–1.17)	0.961	——	——
Smoking, %	2.09 (0.83–5.23)	0.117	——	——
Hypertension, %	0.98 (0.39–2.48)	0.964	——	——
Diabetes, %	0.85 (0.31–2.34)	0.757	——	——
Cr, μmol/L	1.00 (0.99–1.01)	0.496	——	——
Genotype distribution				
CT	0.64 (0.25–1.63)	0.350	——	——
TT	0.61 (0.07–5.48)	0.663	——	——
CT + TT	0.64 (0.26–1.57)	0.327	——	——
BNP, pg/mL	1.00 (1.00–1.00)	0.061	1.00 (1.00–1.00)	0.061
Hb, g/L	0.98 (0.95–1.01)	0.221	——	——
PLT,/L	1.00 (0.99–1.01)	0.874	——	——
LDL‐C, mmol/L	0.80 (0.45–1.40)	0.428	——	——
ALT, U/L	0.99 (0.94–1.05)	0.756	——	——
AST, U/L	0.96 (0.91–1.01)	0.119	——	——

### 3.3 Analysis of Bleeding, All‐Cause Mortality, and Revascularization

Bleeding Events: No significant association was observed between genetic polymorphisms (rs776746 or rs2242480) and bleeding events.

Coronary Revascularization: For the rs776746 locus, the CC genotype may be associated with an increased risk of revascularization, whereas the CT/TT genotypes might confer a protective effect, potentially mediated through improved drug metabolism reducing restenosis. Triple‐vessel disease (TVD) emerged as a strong independent risk factor, exerting substantially stronger effects than genotype. These findings suggest that revascularization risk results from the joint contributions of drug metabolism efficiency (genetically influenced) and coronary anatomical characteristics, necessitating integrated risk prediction incorporating imaging assessments.

All‐Cause Mortality: No direct association was found between genotype and all‐cause mortality.

### 3.4 Generalized Multifactor Dimensionality Reduction (GMDR) Modeling

**FIGURE 2:**
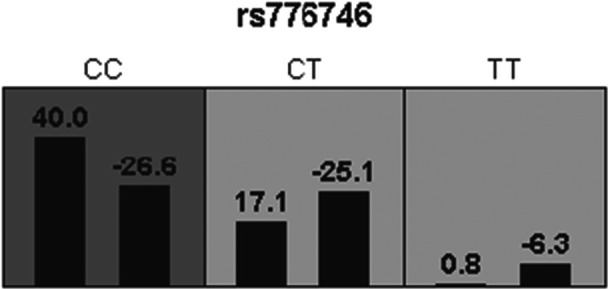
Diagram of first‐order gene‐gene interaction model for rs776746 locus.

The GMDR Analysis for Dyspnea: GMDR analysis demonstrated that the independent model for the rs776746 locus exhibited stable predictive performance for dyspnea risk within 1 year of ticagrelor therapy. This was evidenced by balanced accuracies of 0.6157 in the training set and 0.6079 in the testing set, with perfect cross‐validation consistency (10/10). Statistical significance was confirmed (*p* = 0.0010), indicating a significant association and suggesting its potential as an independent genetic predictive biomarker(Figure 2; Table 5).

**TABLE 5:** Gene‐gene interaction model for ticagrelor‐induced dyspnea identified by generalized multifactor dimensionality reduction (GMDR) analysis.

Model	Balanced accuracy (Training set)	Balanced accuracy (Testing set)	Cross‐validation consistency (CVC)	Significance test (*p* value)
rs776746	0.6157	0.6079	10/10	10 (0.0010)
rs776746‐rs2242480	0.6221	0.5693	10/10	8 (0.0547)

**FIGURE 3:**
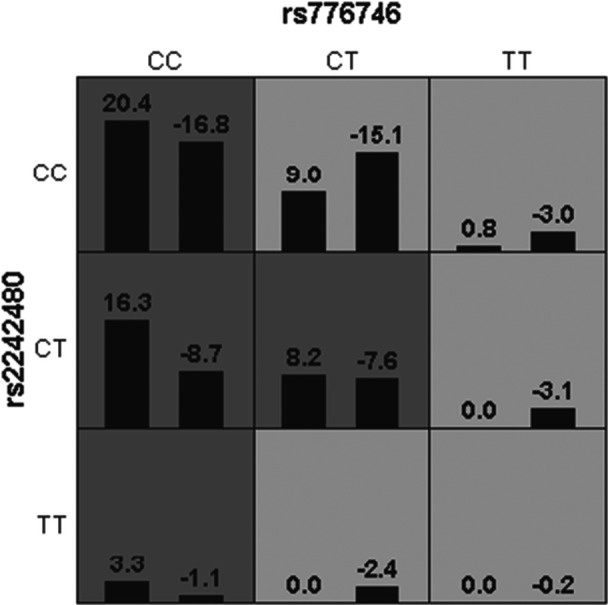
Graphical representation of the second‐order gene‐gene interaction model for dyspnea at the rs776746 locus Note: High‐risk genotype combinations are indicated by dark shading, low‐risk combinations by light shading, and no‐risk combinations by unshaded cells. The color of each cell is determined by its score. Dark shading indicates cells where the score exceeds the threshold value. Light shading indicates cells where the score does not exceed the threshold value. White indicates cells with no data (empty cells). Within each cell: The left bar represents the sum of positive scores. The right bar represents the sum of negative scores. Since this study established a positive correlation between dyspnea and reduced‐metabolism genotypes, we designate: Dark‐shaded cells as high‐risk genotypes. Light‐shaded cells as low‐risk genotypes.

Interaction Model Performance: In contrast, the interaction model incorporating both rs776746 and rs2242480 showed a slightly higher balanced accuracy (0.6221) and sensitivity (0.7767, indicating strong positive sample identification) in the training set. However, it displayed significantly lower specificity (0.4675, reflecting poor negative sample discrimination). More critically, its performance substantially declined in the testing set (balanced accuracy = 0.5693), and the result did not reach statistical significance (*p* = 0.0547) (Figure 3).

Interpretation of Interaction Results: These findings suggest the interaction model carries a risk of overfitting. The high training sensitivity may stem from capturing noise rather than true biological signal. The performance drop in the testing set and the borderline P‐value indicate that the synergistic effect between these two loci likely has limited clinical relevance in real‐world settings. Further validation in larger cohorts is warranted to confirm any potential biological significance.

## 4 Discussion

Dyspnea: The rs776746 (CYP3A5) locus demonstrated significant association with dyspnea risk: carriers of the CT genotype (OR = 0.45, 95% CI = 0.26–0.80, *p* = 0.006) and TT genotype (OR = 0.09, 95% CI = 0.01–0.65, *p* = 0.017) exhibited 55% and 91% reduced risk, respectively. The combined CT/TT genotypes (representing intermediate/rapid metabolizer phenotypes) showed a 63% risk reduction (OR = 0.37, *p* = 0.001), indicating that functional CYP3A5 activity protects against ticagrelor‐associated dyspnea, likely by enhancing drug clearance and reducing systemic accumulation.

Bleeding Events: Neither locus reached statistical significance for bleeding outcomes. However, with only 13 events in the rs776746 cohort and six in the rs2242480 cohort, the analysis was underpowered due to the limited number of events.

Coronary Revascularization: While rs776746 CT/TT genotypes showed a non‐significant trend toward reduced revascularization risk in multivariable analysis (adjusted *p* > 0.05), triple‐vessel disease (TVD) was a far stronger predictor (HR = 3.12, *p* < 0.001), underscoring the predominant role of anatomical complexity.

All‐Cause Mortality: No direct genetic associations were identified. Independent predictors included hypertension (HR = 2.1, *p* = 0.01), female sex (HR = 1.8, *p* = 0.03), smoking (HR = 1.9, *p* = 0.02), and elevated B‐type natriuretic peptide (BNP) (HR = 3.4, *p* < 0.001).

Pharmacokinetic alterations mediated by CYP3A5 genetic variation establish a pathophysiological cascade for ticagrelor‐induced dyspnea. As the primary metabolic enzymes of ticagrelor, CYP3A4/5 polymorphisms—particularly the rs776746 variant (CYP3A53)—critically regulate drug disposition kinetics. The CC genotype (poor metabolizer phenotype) causes functional CYP3A5 deficiency, reducing drug clearance and sustaining elevated plasma concentrations, thereby promoting ticagrelor accumulation in respiratory tissues. This accumulation triggers dyspnea through three interconnected mechanisms: (1) Direct sensory activation, where high drug concentrations stimulate pulmonary C‐fiber terminals via Transient Receptor Potential Vanilloid 1 (TRPV1) receptors, inducing neurogenic inflammation [6]; (2) Mediator release, inducing mast cell degranulation with histamine and leukotriene B4 (LTB4) release, provoking bronchial smooth muscle contraction [7]; and (3) Vascular dysregulation, enhancing bradykinin‐mediated vascular permeability that culminates in airway mucosal edema [8]. This pathophysiological mechanism critically explains the clinical observation that CC genotype carriers exhibited a 2.3‐fold higher incidence of dyspnea compared to CT/TT genotypes (*p* < 0.001), consistent with previous reports [5].

Beyond pharmacokinetic effects, CYP3A4/5 genetic polymorphisms may indirectly mediate respiratory adverse reactions by modulating endogenous immune homeostasis, particularly from the perspective of inflammatory or immunoregulatory pathways. As multifunctional cytochrome P450 enzymes, CYP3A4/5 are not only involved in drug metabolism but also catalyze the biosynthesis and degradation of steroid hormones (e.g., cortisol) and arachidonic acid derivatives (e.g., epoxyeicosatrienoic acids, EETs) [9, 10]. Altered enzyme activity resulting from the rs776746 variant may trigger a triple cascade effect: Steroid Metabolic Imbalance: Impaired cortisol synthesis attenuates the anti‐inflammatory effects of glucocorticoids, leading to the accumulation of aldosterone precursors that promote sodium/water retention and exacerbate airway edema [11]. Lipid Mediator Dysregulation: Reduced EETs generation diminishes their bronchodilatory and anti‐inflammatory functions [12], while accumulation of 20‐hydroxyeicosatetraenoic acid (20‐HETE) activates the NF‐κB pathway, promoting the release of TNF‐α and IL‐8 [13]. Immune Cell Polarization: Dysregulation of steroid/lipid mediators induces preferential Th2 cell differentiation [14]. This subsequently upregulates IL‐4/IL‐13 expression, causing mast cell sensitization and mucus hypersecretion [15]. This disruption of the “metabolite‐immune” axis ultimately contributes to airway hyperreactivity, enhanced neurogenic inflammation, and increased vascular permeability. These mechanistic findings are highly congruent with clinical observations of genotype‐dependent dyspnea risk (CC genotype associated with a 2.1‐fold increased risk, *p* = 0.003).

Regarding autonomic nervous system dysregulation, studies suggest that genetic polymorphisms may contribute to interindividual variations in autonomic function regulation [16]. Specific genotypes might increase susceptibility to dyspnea by influencing vagal tone or airway smooth muscle responsiveness.

Currently, the precise mechanism underlying ticagrelor‐associated dyspnea remains unclear, with several hypotheses proposed. These include the adenosine accumulation theory [17, 18], the neuronal P2Y12 receptor inhibition theory [19, 20], and mechanisms resembling transfusion‐related acute lung injury (TRALI) [21, 22]. This study further investigated potential mechanisms for ticagrelor‐associated dyspnea and specifically confirmed the absence of statistically significant differences in bleeding, all‐cause mortality, or revascularization events related to pharmacokinetics.

The negative association for CYP3A4 rs2242480 must be interpreted with caution due to limited power to detect moderate genetic effects (OR < 2.0). Future larger studies are needed to definitively exclude a role for this variant.

### 4.1 Limitations

Sample Size Constraints: The statistical power for certain analyses was limited. This was particularly evident for: The exploratory analysis of CYP3A4 rs2242480 (*n* = 136). ﻿Adverse events with low observed incidence rates (e.g., bleeding events, *n* = 13 for rs776746 cohort). This limits definitive conclusions for this locus, and larger studies are needed. Restricted Genetic Scope: The analysis focused exclusively on CYP3A4 rs2242480 and CYP3A5 rs776746 polymorphisms. Polymorphisms in other genes potentially influencing ticagrelor metabolism (e.g., other CYP isoforms, transporters) or mechanisms underlying adverse reactions (e.g., adenosine signaling, inflammatory pathways) were not investigated. Absence of Pathway Interaction Analysis: The study did not evaluate potential epistatic effects between the studied SNPs and variants in other metabolic or pharmacodynamic pathways. This limits our understanding of potential synergistic or antagonistic genetic interactions contributing to adverse drug reactions.

## 5 Conclusion

In summary, this study provides evidence for a robust association between the CYP3A5 rs776746 polymorphism and ticagrelor‐associated dyspnea, with CC genotype carriers exhibiting a significantly elevated risk (2.3‐fold increase). This finding offers a foundation for clinical decision‐making in personalized antiplatelet therapy: Clinical Practice: Patients with the CC genotype warrant intensified monitoring for respiratory symptoms. Assessment of metabolic status, potentially incorporating therapeutic drug monitoring (TDM), is recommended, with consideration of switching to an alternative antithrombotic regimen if necessary. Technology Translation: Development and integration of rapid rs776746 genotyping technologies (e.g., CRISPR‐Chip) into the acute coronary syndrome (ACS) management pathway could enable genotype‐guided precision prescribing. Mechanistic Intervention: Targeting the TRPV1 pathway (e.g., with antagonists like SB‐705498) represents a potential strategy to prevent drug accumulation‐mediated neurogenic inflammation.

Although the rs776746/rs2242480 interaction model carries a risk of overfitting (test set accuracy: 0.569, *p* = 0.055), the single‐gene effect of rs776746 remains robust, and its predictive performance surpasses that of conventional clinical parameters. Future prospective randomized trials are required to validate the clinical benefit of genotype‐guided management. Furthermore, exploring integrated decision models combining genotyping with dynamic drug concentration monitoring will be essential to ultimately optimize the risk‐benefit ratio of ticagrelor therapy.

## Conflicts of Interest

The authors declare no conflicts of interest.

## Data Availability

The data that support the findings of this study contain potentially identifiable patient information and are not publicly available due to ethical restrictions and patient privacy laws in China. However, de‐identified data are available from the corresponding author (Dr. Feng Chen, E‐mail: 522808393@qq.com) upon reasonable request and subject to approval by the Ethics Committee of ChangHai Hospital, Naval Military Medical University.
